# Enrichment and Diversification of the Wheat Genome via Alien Introgression

**DOI:** 10.3390/plants13030339

**Published:** 2024-01-23

**Authors:** Jeffrey Boehm, Xiwen Cai

**Affiliations:** 1 USDA-ARS, Wheat, Sorghum & Forage Research Unit, Lincoln, NE 68583, USA; jeffrey.boehm@usda.gov; 2 Department of Agronomy and Horticulture, University of Nebraska, Lincoln, NE 68583, USA

**Keywords:** wheat, genetic variability, wild species, wild relatives, chromosome engineering, meiotic homoeologous recombination, alien introgression

## Abstract

Wheat, including durum and common wheat, respectively, is an allopolyploid with two or three homoeologous subgenomes originating from diploid wild ancestral species. The wheat genome’s polyploid origin consisting of just three diploid ancestors has constrained its genetic variation, which has bottlenecked improvement. However, wheat has a large number of relatives, including cultivated crop species (e.g., barley and rye), wild grass species, and ancestral species. Moreover, each ancestor and relative has many other related subspecies that have evolved to inhabit specific geographic areas. Cumulatively, they represent an invaluable source of genetic diversity and variation available to enrich and diversify the wheat genome. The ancestral species share one or more homologous genomes with wheat, which can be utilized in breeding efforts through typical meiotic homologous recombination. Additionally, genome introgressions of distant relatives can be moved into wheat using chromosome engineering-based approaches that feature induced meiotic homoeologous recombination. Recent advances in genomics have dramatically improved the efficacy and throughput of chromosome engineering for alien introgressions, which has served to boost the genetic potential of the wheat genome in breeding efforts. Here, we report research strategies and progress made using alien introgressions toward the enrichment and diversification of the wheat genome in the genomics era.

## 1. Introduction

Wheat is a major food grain source for humans worldwide. However, wheat production has been continually threatened by various biotic and abiotic stresses, such as new disease pathogens/pests and climate variability/change. Hence, there is a constant need to boost the genetic potential of wheat in sustaining/improving wheat production under emerging threats. Over the last few decades, the genetic gain for wheat production has declined due to the draining of the primary gene pool in wheat breeding [1,2]. Climatic changes further threaten wheat production in traditional geographic regions, with evidence of wheat yield reaching an upper yield plateau [2]. The limited genetic variability of the wheat genome used by breeding programs to develop new cultivars has increasingly become a bottleneck of wheat improvement. Thus, there is an urgent demand for wheat breeding programs to enrich and diversify the wheat genome using wheat’s many wild relatives.

**Figure 1:**
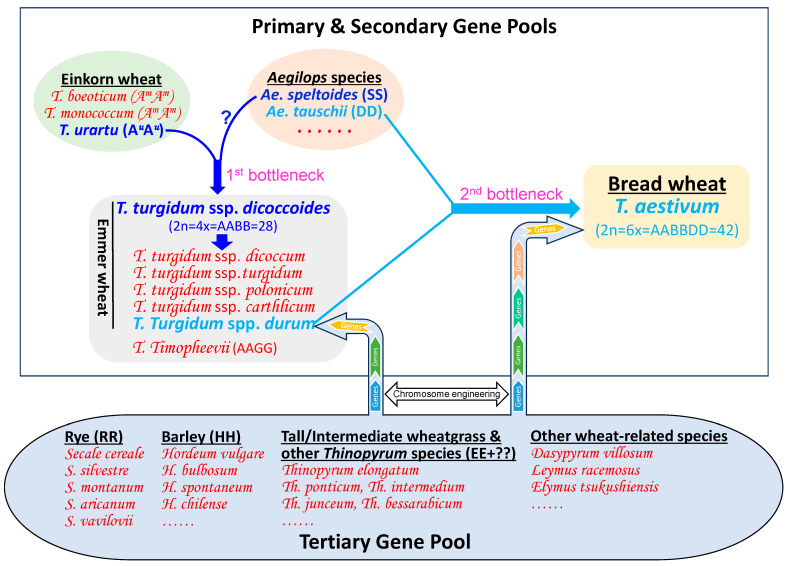
Illustration depicting the origin, evolution, bottlenecks, gene pools, and alien introgression in wheat.

Wheat has a large secondary and tertiary gene pool, including wheat ancestors and relatives that share homologous or homoeologous genomes with wheat (Figure 1). Tremendous genetic variability and diversity have been exploited and identified in the ancestral species and relatives of wheat, which represent an invaluable gene source for wheat improvement. The genetic variability of ancestral species, which share a genome with wheat, can be introduced into the wheat genome via meiotic homologous recombination [3]. Gene introgression from the relatives with genomes homoeologous to the wheat genome into wheat can be achieved by inducing meiotic homoeologous recombination [3]. An alternative approach to transferring genes from non-homologous genomes of the relatives to wheat is through inducing chromosomal damage via irradiation and chemical treatments such as ethyl methanesulfonate (EMS) mutagenesis [4,5]. Recent advances in genomics and the availability of reference genome sequences have dramatically improved the efficacy and throughput of chromosome engineering in alien introgression [3,6,7,8,9,10,11]. Herein, we aim to present research progress and challenges in the enrichment and diversification of the wheat genome.

## 2. Elasticity of the Wheat Genome

Wheat, including common wheat (*T. aestivum* L., 2n = 6x = 42, AABBDD) and durum wheat (*T. turgidum* L. ssp. durum, 2n = 4x = 28, AABB), is an allopolyploid with two homoeologous subgenomes (A and B) in durum and three homoeologous subgenomes (A, B, and D) in common wheat. They originated from interspecific hybridization of two or three diploid ancestors followed by spontaneous chromosome doubling [12,13,14,15,16,17]. The wild einkorn wheat species *T. urartu* (2n = 2x = 14, genome AA) contributed subgenome A to durum wheat, whereas diploid goatgrass species *Ae. tauschii* (2n = 2x = 14, genome DD) contributed subgenome D to common wheat [12,14,15,18]. It remains unclear which ancestor contributed subgenome B, although *Ae. speltoides* has been considered a possible candidate [13,19,20,21]. Emmer wheat (*T. turgidum* L., 2n = 4x = 28, AABB) contributed both subgenomes A and B to common wheat [19,20,21,22]. Homoeologous chromosomes from each of the three subgenomes can genetically compensate for one another, which provides tremendous flexibility in chromosome engineering efforts to enrich and diversify the wheat genome [3,6,7,10,11,22,23,24,25,26,27,28,29,30,31,32,33,34,35,36,37,38,39,40,41,42]. Therefore, the wheat genome can be enriched and diversified by regular meiotic homologous recombination and inducing meiotic homoeologous recombination and physical modifications as well. 

Over the last couple of decades, new genomic technologies and resources available in models and wheat ancestors have dramatically enhanced the characterization and understanding of the large polyploid genome of wheat [43,44]. In recent years, rapid progress has been made toward a better understanding of the structure, function, and evolution of the wheat genome [17,45,46,47,48]. Tremendous genomic resources, such as the Chinese Spring, Svevo, and Zavitan reference genomes, respectively, and other tools specific to wheat and its relatives have been developed and utilized in wheat genomic studies, molecular breeding, and chromosome engineering [3,8,9,49,50]. More recently, a genomics-enabled chromosome engineering pipeline has been developed and optimized for alien introgressions and meiotic homoeologous recombination-based genome studies in wheat and its relatives. A large set of wheat germplasm involving various wheat–alien species’ homoeologous recombination and different genes were developed. Some of them have been utilized in wheat breeding and deployed in wheat varieties [3,4,5,6,7,8,9,10,11,21,22,23,24,25,26,27,28,29,30,31,32,33,42]. 

## 3. Meiotic Homologous Recombination-Based Introgression from the Ancestral Species and Relatives into Wheat

Wheat has three diploid ancestors, including einkorn wheat species *T. urartu* (AA), *Aegilops* species *Ae. tauschii* (DD), and possibly *Ae. speltoides* (SS or BB?), as well as the tetraploid ancestor emmer wheat *T. turgidum* L. ssp. *durum* (AABB). Each of these ancestors has many siblings and relatives that were not involved in the original hybridization events and therefore were left out of the wheat lineage. Wide genetic diversity has been found in the ancestors and their siblings/relatives, but most were not involved in the wheat evolutionary lineage due to the first and second bottlenecks (Figure 1). Despite that, these ancestors and their siblings/relatives generally share one or two homologous genomes with wheat, allowing their genetic diversity to be introduced into the wheat genome via meiotic homologous recombination during routine crossing efforts undertaken by wheat breeding programs [3,15,17,22,28,30]. Some natural hybridization barriers may exist between the ancestral species/relatives and wheat (durum and bread wheat), but generally, these can be easily overcome using the immature embryo rescue technique [51].

Einkorn wheat, including *T. urartu* (A^u^A^u^) and *T. monococcum* (A^m^A^m^), contains an A genome with homology to the A genome of both durum and bread wheat, respectively. Both diploid einkorn wheat species exhibit high genetic variability, which has been exploited to enrich and diversify the wheat A genome via genome-wide introgression and individual gene transfer [50]. For example, large-scale disease-screening efforts detected high frequencies of resistance to stem rust in *T. urartu* (93.0% of 205 accessions) and *T. monococcum* (78.7% of 1061 accessions) [52]. Many genes for resistance to the diseases of stem, leaf and yellow rust, cereal cyst nematode, root lesion nematode, Karnal bunt, powdery mildew, and eyespot have been identified from einkorn wheat, and some of them have been incorporated into wheat varieties by meiotic recombination [53,54,55,56,57,58,59,60]. Generally, this homologous recombination-based introgression is performed by first crossing the diploids to wheat varieties with subsequent backcrossing to recover the wheat genetic background of the recurrent parent. In addition, unique allelic variants have been found for the high- and low-molecular-weight glutenin subunits in *T. urartu*, which have been useful in breeding for targeted end-use quality traits [61]. Moreover, Austin et al. [62] found that *T. monococcum* had higher rates of photosynthesis than common bread wheat. Thus, this einkorn wheat species could harbor genes that may lead to wheat grain yield improvement. 

The wheat D genome donor *Ae. tauschii* (DD), which is placed under the genus *Aegilops*, has been documented as a rich gene reservoir for wheat improvement [63]. Additionally, other *Aegilops* species also contain a D genome [64]. The D genome-originated genes of *Ae. tauschii* can be incorporated into common wheat via homologous recombination with its D genome and by the production of hexaploid synthetic wheat (2n = 6x = 42, genome AABBDD) [65]. Genes for resistance to many diseases and insects, including rusts, powdery mildew, *Septoria nodorum*, *Septoria tritici*, Cyst nematode, Hessian fly, and greenbug, and those for other agronomically important traits, have been identified from *Ae. tauschii* [63]. Some of the *Ae. tauschii*-derived genes have already been transferred to wheat via meiotic recombination [54,63,64,65,66,67,68,69] and synthetic wheat production [63,65,70,71,72,73,74,75,76], offering direct proof of their high utility.

Moreover, there have also been concerted efforts to facilitate the reciprocal exchange or translocation of portions of chromosomes of the D genome of *Ae. tauschii* to complement or replace portions of tetraploid *T. turgidum* L. ssp. *durum* wheat’s A or B subgenomes, respectively, for the specific purpose of improving end-use quality traits that define hard and soft wheat consumer preferences across the globe. In this case, the translocated chromosomes will typically contain genes and distinct haplotypes that are absent in their homoeologous counterparts. Because the D genome of *Ae. tauschii* contains many unique genes that are critically important for not only the characterization of wheat’s distinct market classes, i.e., hard and soft, but also their mechanical milling properties that influence the generation of flours used commercially for the development of end-use quality food products, both the grain *Hardness* (*Ha*) locus and the tightly linked *Puroindoline* (*Pin*) alleles, which condition the PIN A and PIN B proteins on chromosome 5D, in addition to the gluten *Glu-D1* locus and associated high-molecular-weight (HMW) glutenin subunits, which are conditioned by the *Glu-D1d* (Dx5 + Dy10 subunits, optimum for hard wheat end-use quality) or *Glu-D1a* (Dy2 + Dy12 subunits, optimum for soft wheat end-use quality) alleles, respectively, were obvious targets used to transform the capabilities of durum wheat’s end-use quality. For the former, a 5D-5B reciprocal terminal translocation containing the *Ha* locus and *Pin* alleles was moved to the Italian spring durum cultivar Svevo via *ph1b*-mediated homoeologous recombination [40], thereby drastically softening the kernel texture and creating a new wheat market class: Soft Durum. For the latter, a 1D-1A terminal translocation containing the *Glu-D1d* allele at the *Glu-D1* locus from hard red spring wheat cultivar “Len” possessing the Dx5 + Dy10 HMW glutenin subunits was moved to durum wheat cultivars Langdon and Renville [31]. These two examples offer further proof of the high utility of Ae. tauschii-derived genes not only for disease resistance but also for improving durum wheat’s end-use quality.

The tetraploid ancestor (*T. turgidum* L. ssp. *durum*) of common wheat has several related subspecies under *T. turgidum* L., including *T. turgidum* L. ssp. *dicoccoides*, *T. turgidum* L. ssp. *dicoccum*, *T. turgidum* L. ssp. *turgidum*, *T. turgidum* L. ssp. *polonicum*, and *T. turgidum* L. ssp. *carthlicum*. Each ancestor contains the A and B genomes at different domestication stages compared to those in durum and common wheat, respectively [17]. Another tetraploid species in the *Triticum* family, *T. timopheevii*, contains the A genome with divergence from durum and common wheat’s A genome. Many of these tetraploid species have been found to harbor unique genes that durum and common wheat do not have, including genes for elevated grain protein content [77] and resistance to Fusarium head blight (FHB) [78,79], powdery mildew, cereal rusts, and Hessian fly [63,80,81,82,83,84,85]. Many of them have been deployed in the wheat varieties using traditional meiotic homologous recombination-based breeding schemes, which has greatly broadened the genetic variability of the wheat A and B genomes.

## 4. Introduction of an Alien Genome or Individual Chromosomes into Wheat 

Both durum and common wheat are polyploids with two and three homoeologous subgenomes, respectively, that compensate for each other. The polyploid nature of the wheat genome enables wheat to tolerate aneuploidy and to withstand the introduction of alien chromatin, offering a path for the enrichment and diversification of the wheat genome through the introduction of genes from wheat-related alien species into wheat by chromosome additions, substitutions, and translocations [22,28].

**Figure 2:**
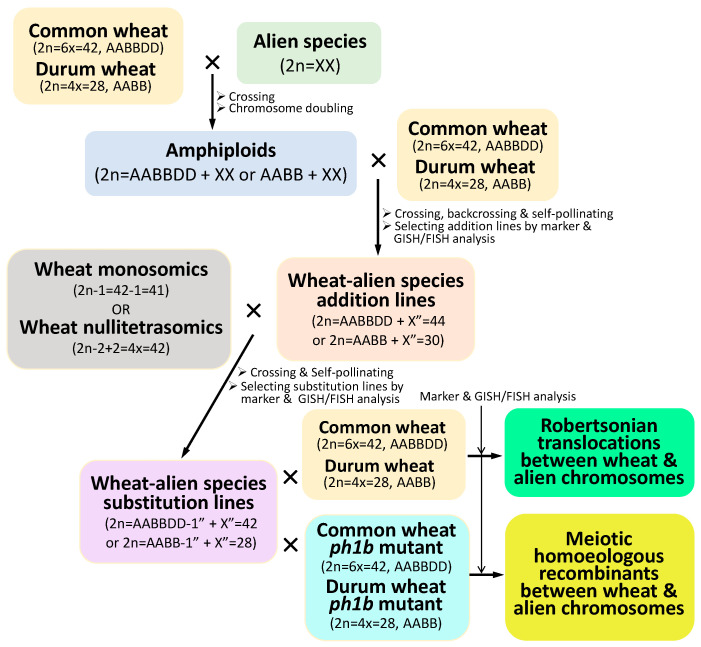
Introduction of alien chromosomes and chromosomal segments into wheat.

A genome or individual chromosomes of an alien species related to wheat can be added to the wheat genome via interspecific hybridization. The intact or partial genome of an alien species can be combined with the wheat genome as amphiploids by hybridizing wheat to the alien species and subsequent chromosome doubling, which is the case for triticale, the generation of hexaploid synthetic wheat, and many other amphiploids (Figure 2) [27]. Generally, amphiploids combine the characteristics of both wheat and alien species, but that combination is not equivalent to a simple one-plus-one chromosomal addition due to intergenomic interactions, polyploidization-related genome modifications, or epigenetic changes [86,87,88,89,90]. For example, an artificial wheat–alien species amphiploid can be developed into a new species, such as triticale (×*Triticosecale*), that combines and presents elements of both wheat and rye (*Secale cereale*) genomes. Triticale is the first man-made cereal plant species that has been widely grown for grain and forage production worldwide. Over the last couple of decades, perennial wheat has also been developed from wheat-perennial grass amphiploids and derivatives to preserve soils from erosion, in addition to more sustainable grain production [91,92]. However, many of the wheat–alien species amphiploids and derivatives cannot be grown directly as a crop because the addition of an alien genome or chromosomes to the wheat genome brings favorable as well as unfavorable genes into wheat, making them unsuitable for direct production. Notwithstanding, the wheat–alien species amphiploids and derivatives have been commonly used as bridging materials for gene transfer from the alien species to wheat (Figure 2) [22,28,93,94,95]. In addition, individual chromosomes of an alien species can be introduced into wheat by producing wheat–alien species addition lines, a method that dissects the genome of the alien species in the wheat background and identifies the individual alien chromosomes containing the genes of interest for wheat improvement [27,28,96,97,98,99,100,101]. The individual chromosome addition lines can also serve as bridging materials to transfer the genes of interest on a specific alien chromosome to the wheat genome by producing chromosome substitutions and translocations (Figure 2) [102].

Another approach to introducing individual chromosomes into wheat is to produce chromosome substitution lines in which a wheat chromosome is substituted by a homoeologous counterpart of an alien species. The wheat–alien species substitution lines are generally more stable than their corresponding addition lines in terms of meiotic transmission of the alien chromosomes in the wheat genomic background if the critical chromosomes involved in the substitution well compensate for each other. Disomic substitution lines have been developed from many alien species, including rye, barley, *Thinopyrum elongatum*, and *Aegilops speltoides* [27,102,103,104]. Out of the wheat–alien substitutions, the wheat–rye 1R(1B) substitution is the most prevalent wheat–alien chromosome substitution deployed in the breeding of new wheat varieties. The introduction of rye chromosome 1R into wheat via substitution has led to the development of many early wheat varieties in Eastern Europe and later in other countries worldwide due to the presence of multiple genes on 1R for resistance to diseases, tolerance of insects, and enhanced yield potential, especially in soils with low fertility [25,105,106,107,108,109]. Furthermore, the wheat–alien species substitution lines are ideal intermediate materials used to induce meiotic homoeologous recombination [3,6,10,11,21,41,42] and Robertsonian translocations [110] (Figure 2). The chromosomes involved in the substitution can be specifically targeted for meiotic homoeologous recombination in the near-isogenic backgrounds [3,10,11,41,42].

## 5. Meiotic Homoeologous Recombination-Based Alien Introgression from Relatives of Wheat

Barley (*Hordeum vulgare,* 2n = 2*x* = 14, HH) and rye (*S. cereale,* 2n = 2*x* = 14, RR) are examples of two cultivated cereal relatives of wheat. The barley genome H and rye genome R are homoeologous to the wheat sub-genomes A, B, and D, respectively. In addition, many wild grass species contain genomes homoeologous with one or more of the wheat sub-genomes. The chromosomes of wheat-related species generally do not synapse and recombine with wheat chromosomes during meiosis in the presence of pairing homologous gene *Ph1*, a primary gene responsible for the allopolyploid behavior of wheat species [111,112]. Deletion of the genomic region harboring *Ph1* on wheat chromosome 5B led to the generation of the *ph1b* mutant in hexaploid common wheat [112] and *ph1c* mutant in tetraploid durum wheat [113]. The loss of *Ph1* function promotes meiotic synapsis and the recombination of wheat chromosomes with their homoeologous counterparts of the alien species. More recently, Gyawali et al. [114] mapped the deletion breakpoints of the *ph1b* mutation and developed DNA markers specific for both alleles, which has improved the efficacy and throughput of the *ph1b* mutant in the homoeologous recombination-based alien introgression pipeline. In addition, two unlinked genes that inhibit Ph function, designated PhI and *Su1-Ph1*, were identified from *Ae. specltoides* and transferred to wheat. These two genes inhibit the activity of the *Ph1* gene in wheat, further promoting meiotic homoeologous recombination for alien introgressions [115,116,117,118].

Recent advances in the genomics of wheat, its ancestors, and relatives have provided numerous genomic resources and tools to advance chromosome-related research, including next-generation DNA sequencing and sequenced reference genomes, genome-wide single nucleotide polymorphisms (SNPs) libraries, and SNP-based polymerase chain reaction (PCR) markers. A large set of chromosome-specific SNP-based PCR markers, such as kompetitive allele-specific PCR (KASP) markers, PCR allelic competitive extension (PACE) markers, and semi-thermal asymmetric reverse PCR (STARP) markers, have been developed based on sequence alignments of reference genomes of wheat, its ancestors, and relatives [8,119,120]. Chromosome-specific markers have proven extremely useful in targeted alien introgressions and dramatically improve the efficacy and throughput of meiotic recombination-based gene transfer from alien species to wheat. They have been widely utilized in chromosome engineering-based alien introgression and marker-assisted selection of alien genes in wheat breeding [3,6,7,10,11,35,41,42]. 

The barley genome (HH) has relatively low homoeology with the wheat genome in comparison to the genomes of some other wheat-related species. Wheat–barley homoeologous recombinants have been produced by inactivating *Ph1* gene function using the *ph1b* mutant, *Ph^I^*, and 5B nullisomics, the same as *Su-Ph1* [120,121,122,123,124,125,126,127,128,129,130,131]. Genes for many of barley’s unique traits have been identified, and some of them have been transferred to wheat via meiotic homoeologous recombination, such as the genes for high grain β-glucan (dietary fiber) content on chromosome 7H [120], genes for flowering time/maturity on 7H and 4H [132], and the *Isa* gene for barley bifunctional α-amylase/subtilisin inhibitor (BASI) that may reduce preharvest sprouting of wheat [129]. These barley-to-wheat gene introgressions have introduced barley-specific genes useful for wheat improvement and consequently have diversified the wheat genome. 

Rye is another wheat-related cultivated cereal crop that has been widely exploited for wheat improvement. The most prevalent wheat–rye introgressions in the modern wheat varieties are wheat–rye 1RS·1BL translocations [26,105,133]. Most of the translocations resulted from centric breakage-fusion events instead of meiotic homoeologous recombination due to the limited homoeology between the rye and wheat genomes [134,135,136]. Small wheat–rye 1RS-1BS translocations have been generated from the whole arm 1RS·1BL translocations and other wheat–rye derivatives using the *ph1b* mutant [137,138]. In addition, wheat–rye homoeologous recombination can be induced to generate compensating wheat–rye translocations via loss of *Ph1* function in the wheat × triticale crosses [139,140,141,142,143]. Both centric misdivision and meiotic homoeologous recombination-derived wheat–rye translocations have played a significant role in wheat improvement [133,144,145]. Many wheat–rye chromosome substitutions and translocations have been deployed in wheat varieties with improved resistance/tolerance to various biotic and abiotic stresses in wheat [25,26,27].

A large number of wild grass species are genetically related to wheat, representing an invaluable source of genetic variation to enrich and diversity the wheat genome. Numerous genes for many favorable traits have been incorporated into the wheat genome from wild species, including wild species under the genera of *Aegilops*, *Thinopyrum (Agropyron)*, *Haynaldia* (*Dasypyrum*), and *Leymus*, by inducing meiotic homoeologous recombination using the *ph1b* mutant and the *Ae. speltoides*-derived *Ph1* suppressor gene *Su1-Ph1* [3,6,7,10,11,32,41,42,63,118,146,147,148,149,150,151]. The alien chromosome segments integrated into the wheat genome via homoeologous recombination generally inherit as a single, stable locus because they usually do not recombine with their homoeologous counterparts of wheat in the presence of the *Ph1* gene [111,112,113,114]. In addition, the high number of polymorphisms between the genomes of wheat and alien species makes the development of chromosome-specific markers straightforward [6,120]. Therefore, marker-assisted selection has proven highly effective for the alien chromosome segments and associated genes that have been incorporated into the wheat genome in wheat breeding efforts. Furthermore, the wheat–alien species recombinants can serve as bridging materials to introduce additional genetic variation into the wheat genome from different accessions of the alien species via the homologous recombination of the alien chromosomal segments in the recombinants and their respective homologues from other accessions of the same alien species. This method offers a path that will serve to bridge a constant gene flow from alien species into wheat to enrich and diversify the wheat genome, thereby preventing genetic bottlenecks. 

## 6. Challenges and Perspectives

Many relatives and ancestral species of wheat, including rye, barley, and wild grass species, harbor genes for unique traits wheat does not possess. These collective species represent an invaluable source of genetic variation for wheat improvement with huge potential to open genetic bottlenecks. The meiotic homoeologous recombination-based chromosome engineering pipeline is the most genetically friendly approach for introducing genetic variability from alien species into wheat because the homoeologous chromosomes compensate for each other genetically. There are, however, two major potential challenges in the meiotic recombination-mediated alien introgression. First, the biological barriers of hybridization between wheat and distantly related alien species often could limit access of the alien species for chromosome engineering. To account for this challenge, wide hybridization can be achieved by using tissue culture-based somatic hybridization (protoplast fusion) and embryo rescue techniques [51,152], which has successfully served to bridge access to many alien species for gene transfer in wheat. Second, the genomes of some alien species have low homoeology with the wheat genome, resulting in low meiotic homoeologous recombination between wheat and alien chromosomes, even in the absence and suppression of *Ph1*. In addition, meiotic homoeologous recombination rates may vary with different chromosomes and chromosomal regions. For instance, the pericentromeric region of a chromosome generally has lower recombination frequencies than the distal regions [10,11,21,41]. Additional rounds of secondary and tertiary recombination can be induced between a primary wheat–alien species recombinant and its homoeologous chromosome of wheat in the absence of *Ph1* or using *Ph^I^* and *Su1-Ph1*. The primary wheat–alien species recombinants also share higher levels of homology with wheat homoeologues than the intact alien chromosome, which often enhances meiotic recombination of the alien chromosomal segments in the primary recombinant with their homoeologous wheat counterparts [10,11]. Thus, secondary and tertiary recombination events can increase the accessibility of the chromosomal regions with low recombination frequencies for alien introgression-based enrichment and diversification.

## 7. Conclusions

The polyploid nature and plasticity of the wheat genome are able to withstand chromosome engineering to extend its genetic variability for wheat improvement and to enhance genomic studies in wheat and its relatives. The wheat genome, which has narrow genetic variability due to the limitations of having just three diploid ancestors stemming from two hybridization events in its polyploid evolution, can be enriched and diversified by exploiting its many ancestral species and relatives of wheat. This process can be achieved by performing alien chromosome introgressions through regular meiotic homologous recombination as well as induced meiotic homoeologous recombination. The availability of modern genomics resources and technologies will enhance the efficacy and throughput of alien introgressions in wheat breeding efforts and further genomic studies.

## Data Availability

Not applicable.
